# In Vivo Biodistribution and Efficacy Evaluation of NeoB, A Radiotracer Targeted to GRPR, in Mice Bearing Gastrointestinal Stromal Tumor

**DOI:** 10.3390/cancers13051051

**Published:** 2021-03-02

**Authors:** Christopher Montemagno, Florian Raes, Mitra Ahmadi, Sandrine Bacot, Marlène Debiossat, Julien Leenhardt, Jean Boutonnat, Francesca Orlandi, Donato Barbato, Mattia Tedesco, Catherine Ghezzi, Pascale Perret, Alexis Broisat

**Affiliations:** 1 Laboratoire radiopharmaceutiques biocliniques (LRB), Universite Grenoble Alpes, Inserm, CHU Grenoble Alpes, LRB, 38000 Grenoble, France; cmontemagno@centrescientifique.mc (C.M.); florian.raes@univ-grenoble-alpes.fr (F.R.); mitra.ahmadi@univ-grenoble-alpes.fr (M.A.); sandrine.bacot@univ-grenoble-alpes.fr (S.B.); marlene.debiossat@univ-grenoble-alpes.fr (M.D.); jleenhardt@chu-grenoble.fr (J.L.); Catherine.Ghezzi@univ-grenoble-alpes.fr (C.G.); pascale.perret@univ-grenoble-alpes.fr (P.P.); 2 Therex, Universite Grenoble Alpes, CNRS, CHU Grenoble Alpes, Therex, 38000 Grenoble, France; JBoutonnat@chu-grenoble.fr; 3 Advanced Accelerator Applications, a Novartis Company, 10010 Colleretto Giacosa (TO), Italy; orlandi_francesca@hotmail.com (F.O.); donato.barbato@novartis.com (D.B.); mattia.tedesco@novartis.com (M.T.)

**Keywords:** gastrin releasing peptide receptor, GIST tumor, theragnostic, theranostic, [^177^Lu]Lu-NeoB, NeoBOMB1

## Abstract

**Simple Summary:**

NeoB is undergoing evaluation as a novel theragnostic agent—that is, that it can be employed either for the diagnosis of tumor expressing gastrin-releasing peptide receptor (GRPR) using nuclear imaging, or for the therapy of such GRPR positive tumors using internal radiotherapy. The switch from diagnosis to therapy simply rely on the choice of the radioisotope that is coupled to NeoB. The aim of our study was to investigate—for the first time—the potency of NeoB for tumor therapy once labeled with the beta- emitter Lu-177. This study has been conducted in mice bearing human Gastrointestinal Stromal Tumors (GIST). [^177^Lu]Lu-NeoB was found to accumulate in the tumor, with only minimal retention in off-target organs. Consequently, mice treated with therapeutic doses of [^177^Lu]Lu-NeoB (37MBq/week for three weeks) exhibited tumor regression and therefore long term survival in comparison to the control untreated mice.

**Abstract:**

NeoB is a radiotracer targeting the gastrin-releasing peptide receptor (GRPR), a G-protein–coupled receptor expressed in various cancers. The aim of the present study was to evaluate the biodistribution and efficacy of this new therapeutic agent in Gastrointestinal Stromal Tumors (GIST). Eighty-two SCID mice bearing GIST-882 tumors were employed. [^177^Lu]Lu-NeoB biodistribution was evaluated up to seven days by organ sampling (200 pmol/0.8 MBq, i.v.). For efficacy evaluation, mice received either saline, 400 pmol or 800 pmol of [^177^Lu]Lu-NeoB (37MBq, 1/w, 3 w, i.v.). SPECT/CT imaging was performed at 24 h, and tumor volume was determined up to 100 days. Elevated and specific [^177^Lu]Lu-NeoB uptake was found in the GIST tumor, as demonstrated by in vivo competition (19.1 ± 3.9 %ID/g vs. 0.3 ± 0.1 %ID/g at 4h). [^177^Lu]Lu-NeoB tumor retention (half-life of 40.2 h) resulted in elevated tumor-to-background ratios. Tumor volumes were significantly reduced in both treated groups (*p* < 0.01), even leading to complete tumor regression at the 400 pmol dose. [^177^Lu]Lu-NeoB exhibited excellent pharmacokinetics with elevated and prolonged tumor uptake and low uptake in non-target organs such as pancreas. The potential of this new theragnostic agent in different indications, including GIST, is under evaluation in the FIH [^177^Lu]Lu-NeoB clinical trial.

## 1. Introduction

The Gastrin releasing peptide receptor (GRPR) or bombesin receptor subtype 2 (BB2) is a 7-transmembran G-protein–coupled receptor mainly expressed in pancreas and at lower level in colon, prostate and some regions of the central nervous system [1,2]. Gastrin-releasing peptide (GRP), which belongs to the family of bombesin-like peptide, and binds and triggers GRPR activation, leading to multiple physiological processes such as release of gastrointestinal hormones or smooth cell contraction [3]. GRPR overexpression has been evidenced in a wide spectrum of tumors such as 63 to 100% of prostate, 33 to 72% of breast and 76 to 93% of gastric cancers [4,5,6,7]. Many studies have highlighted that the GRPR pathway is a key actor of cancer progression through increasing proliferation, invasion and migration of cancer cells via autocrine and paracrine pathways [8,9,10].

Several GRPR-directing therapeutic strategies have therefore been developed, including antagonists or monoclonal antibodies, which are ongoing evaluation [10]. GRPR also constitutes a promising biomolecular target in nuclear oncology for a theragnostic approach combining tumor imaging therapy [11]. Such strategy has been shown to be promising in different cancers and a first theragnostic agent directed against SSTR2 positive neuroendocrine tumors has recently been approved by the Food and Drug Administration (FDA) and European Medicines Agency (EMA) [11,12,13]. Several GRPR radioligands have been evaluated [14,15,16,17,18]. Pre-clinical studies showed that antagonists, in comparison to agonists, exhibited higher tumor targeting and better pharmacokinetics profiles [17,19]. Clinical investigations confirmed higher tumor uptake of antagonists in addition to a higher rate of tumor and metastases detection in prostate cancer patients [20]. Moreover, GRPR antagonists display reduced physiologic activity and reduced radioactivity accumulation at physiologic GRPR, leading to fewer side effects than agonists [19].

In the present study we investigate the therapeutic potential of a DOTA-coupled GRPR antagonist called NeoB, previously known as NeoBOMB1. NeoB is derived from the antagonist GRPR peptide SB3 [21]. NeoB was previously labeled with Ga-67, Ga-68, In-111, and Lu-177 [22,23,24]. High affinity, in vivo stability and high tumor to background ratio of radiolabeled-NeoB at early time point were demonstrated in pre-clinical studies of breast and prostate cancer bearing mice [22,23]. First clinical results in prostate cancer patients showed that [^68^Ga]Ga-NeoB rapidly localized in pathologic lesions with high-contrast imaging [24]. GRPR is also highly expressed in gastrointestinal stromal tumor (GIST) patients (~80%), so that NeoB could be a valuable potential drug candidate to treat such tumors that lack effective therapies when resistance to receptor tyrosine kinase (RTK) inhibitors occurs [25,26,27].

In the present study we investigated for the first time the therapeutic potential of [^177^Lu]Lu-NeoB, using mice bearing human GIST.

## 2. Materials and Methods

### 2.1. Cell Line and Culture Conditions

The human gastrointestinal tumor GIST-882 cell line [28] has been provided by S. Schubert (Fraunhofer, Munchen, Germany). GIST-882 were cultured using RPMI 1640 medium supplemented with 15% Fetal Bovine Serum, 100 U/mL Penicillin, 100 µg/mL Streptomycin, 10 µg/mL Gentamycin, and 0.5 µg/mL Amphotericin B. Cells were grown in a humidified atmosphere with 5% CO_2_ at 37 °C.

### 2.2. Radiolabeling of NeoB with Lu-177 and [^177^Lu]Lu-NeoB Stability

^177^Lu^3+^ in aqueous 0.04 M HCl solution was purchased from ITG (Garding, Germany). Chemicals for radiolabeling were of trace metal^®^ grade. Radiochemical purity (RCP) of each radiolabeled solution was determined using a high performance liquid chromatography (HPLC) apparatus (Shimadzu, France) equipped with NaI (Tl) scintillation detectors (LabLogic, UK). The radiolabeling was performed as previously described (25). For the biodistribution sub study, 92 nmol of NeoB was radiolabeled with 370 MBq of ^177^LuCl_3_ and the reaction was then incubated at 95 °C for 7 min. The concentration was then adjusted at 200 pmol/100 µL (0.8 MBq) and a sterilizing filtration was performed. A similar protocol was employed for the therapy sub-study, using 20 nmol of NeoB and 1850 MBq of Lu-177. The concentration was then adjusted at 400 pmol/100 µL (37 MBq). A second solution of 800 pmol per 100 µL (37 MBq) was obtained by the addition of unlabeled NeoB peptide. A 0.22 µm sterile filtration was then performed. Radiochemical purity (RCP) was determined immediately after labeling and at 24 h by radio-HPLC analysis using an analytical reverse-phase column (Symmetry C18, 5 μm, 150 × 4.6 mm). A gradient elution was performed using solvent A (H_2_O, 0.1% TFA) and solvent B (acetonitrile, 0.1% TFA) as mobile phase at a flow rate of 1 mL/min.

### 2.3. Animal Models

All procedures were performed in accordance with the institutional guidelines and approved by the animal care and use committee of Grenoble University (APAFIS#6544-2016082615349282 v8). The study was performed on 6-weeks old male SCID mice (Charles River). A total of 82 mice were used, 42 for the biodistribution sub-study and 40 for the therapy sub-study. Mice were subcutaneously inoculated into the left flank with GIST-882 (2 to 4 × 10^6^) in a 1:1 PBS/Matrigel^®^ (Corning, Corning, NY, USA) mixture.

### 2.4. Biodistribution

Biodistribution of [^177^Lu]Lu-NeoB was evaluated when the tumor volume reached ~200 mm^3^. The biodistribution was evaluated at 7 time points (1 h, 4 h, 4 h + block, 24 h, 48 h, 96 h, and 168 h). The compound was injected intravenously (100 µL/200 pmol/~0.8 MBq per mice). Moreover, an excess of 40 nmol unlabeled NeoB was co-injected in the mice of the 4 h + block condition. At the selected time point, mice were euthanized by CO_2_ inhalation, and the main organs and fluids were sampled and weighed. The activity was determined using a gamma well counter (Wizard^2^ PerkinElmer, Waltham, MA, USA), the results were corrected for background, decay and sample weight, and the data were expressed as a % ID/g (percentage of injected dose per gram of tissue).

### 2.5. Therapeutic Efficacy

Efficacy was evaluated on 40 mice when the tumor volume reached ~100 mm^3^. Control mice were intravenously injected with saline (Control group, *n* = 13), whereas treated mice received either 400 pmol (400 pmol group, *n* = 13) or 800 pmol (800 pmol group, *n* = 12) of 37 MBq of [^177^Lu]Lu-NeoB once a week for 3 weeks. Tumor size was recorded 3 times per week before and after therapy administration and 5 times per week during the 3 weeks of the therapy administration. When a tumor volume exceeding 1500 mm^3^ the animal was removed from the study and euthanized by CO_2_ inhalation. Pancreas was then frozen, 10 µm-thick slices were stained using hemato-eosin saffron (HES) and subsequently analyzed by a histopathologist.

### 2.6. SPECT-CT Imaging

Dual single photon emission computed tomography and x-ray computed tomography (SPECT-CT) imaging were performed in a subset of 6 mice of the therapeutic efficacy sub-study (3 from 400 pmol group and 3 from 800 pmol group). Acquisitions were performed the day after [^177^Lu]Lu-NeoB injection (24 h post injection). SPECT-CT acquisitions were performed using a dedicated camera (nanoscan SPECT/CT, Mediso, Budapest, Hungary). Animals were placed on a pathogen-free animal handling system (MulticellTM, Mediso, Budapest, Hungary) under gas anesthesia (isoflurane 1–2%). Whole body imaging was performed. SPECT duration was of approximately 35 min, and CT acquisition of 5 min (at 35 kvp). Acquisitions were reconstructed with Nucline software using Monte Carlo reconstruction and scatter and attenuation correction, corrected for decay and expressed as % injected dose per gram (% ID/g). Image analysis was performed using Vivoquant software (version 3.5, Invicro, Boston, MA, USA).

### 2.7. Statistics

Effect of in vivo competition with unlabeled NeoB was evaluated using multiple t tests corrected using Holm–Sidak method. The survival between treated groups was analyzed using the log-rank test (*p* < 0.05). Comparison of tumor uptake between 400 pmol and 800 pmol groups by SPECT quantification and comparison of tumor growth between control, 400 pmol and 800 pmol groups were performed using 2-way ANOVA corrected for multi comparison using Sidak test.

## 3. Results

### 3.1. Radiolabeling

**Figure 1:**
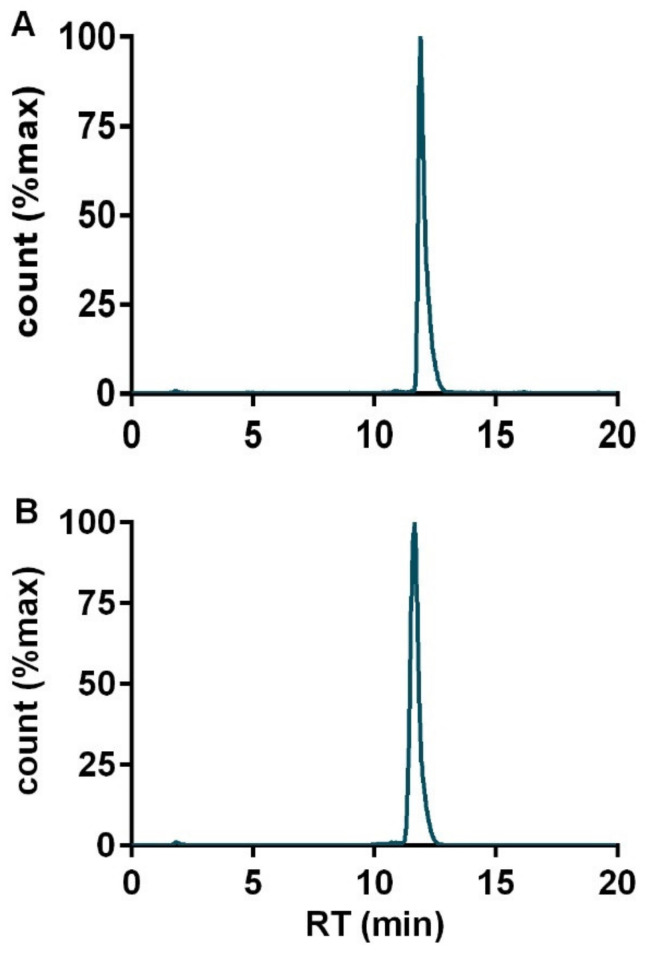
HPLC profiles of [^177^Lu]Lu-NeoB performed (**A**) immediately following radiolabeling and (**B**) 24 h after radiolabeling.

NeoB was successfully radiolabeled with Lu-177. All radioactive preparations show a RCP higher than 98% with a single peak corresponding to [^177^Lu]Lu-NeoB at a retention time of 11 min (Figure 1A). Moreover, [^177^Lu]Lu-NeoB remained stable for 24 h after labeling (Figure 1B).

### 3.2. Biodistribution

**Figure 2:**
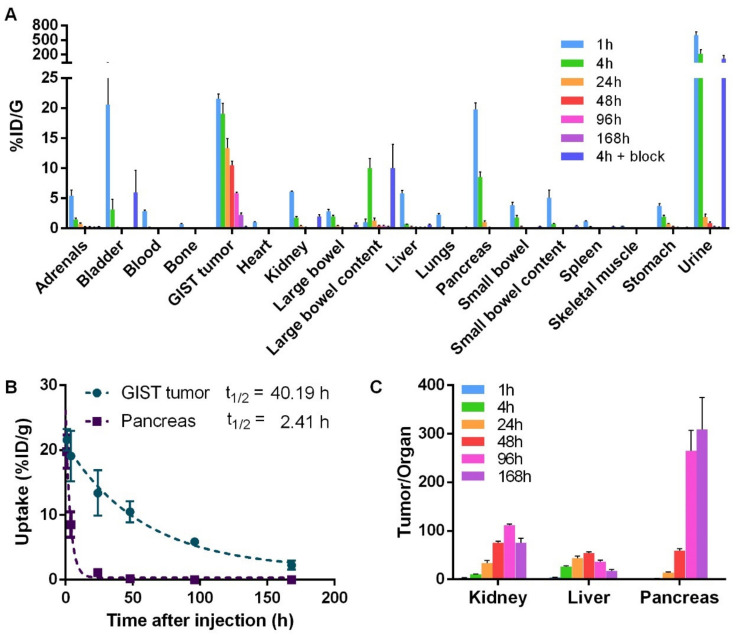
Biodistribution of [^177^Lu]Lu-NeoB on gastrin-releasing peptide receptor (GRPR) expressing tumor bearing mice. (**A**) Biodistributions were performed at 1 h, 4 h, 24 h, 48 h, 96 h, 168 h, and at 4 h when co-injected with an excess of unlabeled NeoB (4 h + block). (**B**) Time activity curve of [^177^Lu]Lu-NeoB in the tumor and pancreas. (**C**) Tumor-to-organ ratio from 1 h to 168 h. Data are represented as mean ± SD.

Biodistributions of [^177^Lu]Lu-NeoB were performed on GIST tumor bearing mice at different time after injection (Figure 2A, Table S1). At 1 h post-injection (pi), [^177^Lu]Lu-NeoB uptake was greater than 2% ID/g in most investigated tissues. Among those tissues, the uptake was greater than 10% ID/g in the urine (600.2 ± 155.3 % ID/g), the bladder (20.6 ± 19.7% ID/g), the GIST-882 tumor (21.6 ± 1.7% ID/g), and the pancreas (19.8 ± 2.6% ID/g). At 4 h pi, [^177^Lu]Lu-NeoB uptake remained greater than 10% ID/g in the urine (215.5 ± 192.9%ID/g), the GIST tumor (19.1 ± 3.9% ID/g) and the large bowel content (10.0 ± 3.5% ID/g). At this 4 h-time point, when co-injected with an excess of unlabeled NeoB, [^177^Lu]Lu-NeoB uptake was significantly reduced in the adrenals, blood, GIST tumor, large and small bowel, pancreas, and stomach (*p* < 0.05). The most profound decrease (> 98%) was observed in the GIST tumor (0.3 ± 0.1 vs 19.1 ± 3.9% ID/g) and the pancreas (0.1 ± 0.0 vs 8.5 ± 2.0% ID/g) (Figure 2A), indicating GRPR specificity. From 24 h to 168 h pi, [^177^Lu]Lu-NeoB uptake was lower than 2% ID/g in all investigated tissues with the exception of the GIST tumor. Indeed, tumor uptake was of 13.4 ± 3.5% ID/g, 10.5 ± 1.6% ID/g, 5.9 ± 0.3% ID/g and 2.5 ± 0.7% ID/g at 24 h, 48 h, 96 h, and 168 h, respectively (Figure 2A, Figure S1). [^177^Lu]Lu-NeoB retention in the tumor was therefore elevated in comparison to that of other tissues such as pancreas with a half-life of 40.2 h (Figure. 2B). Accordingly, tumor-to-kidney, tumor-to-liver, and tumor-to-pancreas ratios were found to be greater than 1 at all investigated time points with a maximum value at 96 h for the tumor-to-kidney ratio (112 ± 5), at 48 h for the tumor-to-liver ratio (54 ± 5), and at 168 h for the tumor-to-pancreas ratio (309 ± 148) (Figure 2C).

### 3.3. Efficacy

**Figure 3:**
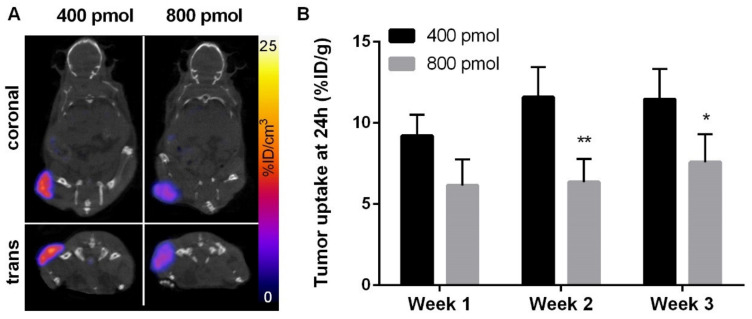
In vivo distribution of [^177^Lu]Lu-NeoB in Gastrointestinal Stromal Tumor (GIST) bearing mice. (**A**) Representative SPECT/CT images acquired 24 h following the injection of 400 pmol (left) or 800 pmol (right) [^177^Lu]Lu-NeoB. SPECT was corrected from background and decay and scaled from 0 to 25% ID/g. (**B**) Quantification of [^177^Lu]Lu-NeoB tumor uptake on SPECT images determined at day 1 after injection on week 1, 2, and 3. Results are expressed in % ID/g. * *p* < 0.05, ** *p* < 0.01 vs. 400 pmol group.

As expected from the previously performed biodistribution evaluation, the strongest [^177^Lu]Lu-NeoB uptake at 24 h was observed by SPECT-CT imaging at the level of the tumor (Figure 3A). Interestingly, [^177^Lu]Lu-NeoB tumor uptake in 400 pmol group mice (~11% ID/g) was found to be higher than that observed in 800 pmol group mice (~7% ID/g) (*p* < 0.05) (Figure 3B).

**Figure 4:**
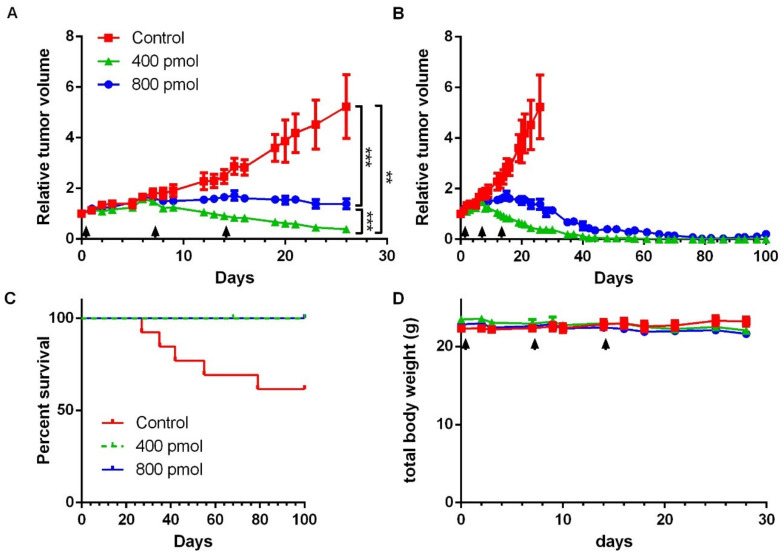
Effect of [^177^Lu]Lu-NeoB on tumor growth. Mice bearing GIST-882 tumor were injected once a week for three weeks with 37 MBq of 400 pmol or 800 pmol of [^177^Lu]Lu-NeoB or with saline (control). Tumor volume was expressed relatively to the volume measured at the time of the first injection (**A**,**B**). At early time points (0–26 days) tumor volume were evaluated on all 40 mice (**A**,**B**). At later time points (28–100 days), 5 control mice out of 13 were removed from the follow up at the time their tumor reached the limit volume size of 1500 mm^3^, so that control group curve is no longer represented on the graphics (**B**) (individual curves are available in Figure S1). (**C**) Percent of survival without reaching the limit tumor volume. (**D**) Total body weight of mice. Data are represented as mean ± SEM. ** *p* < 0.01, *** *p* < 0.001.

**Figure 5:**
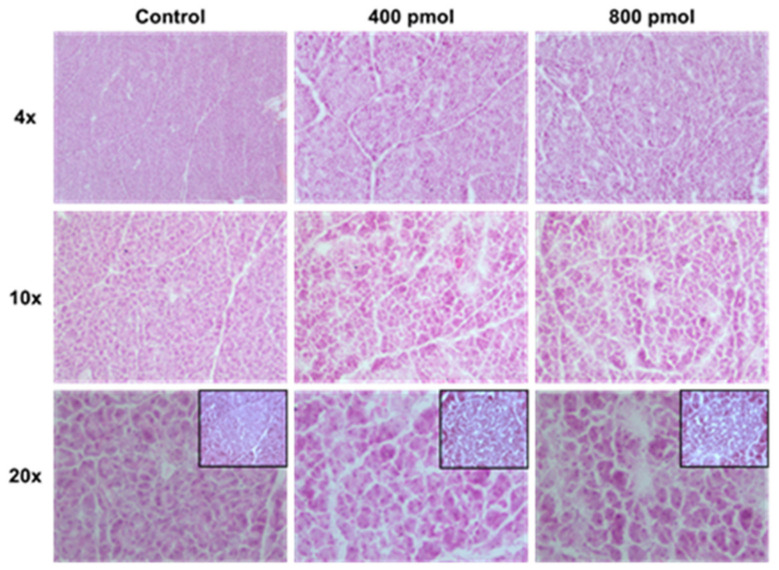
Pancreas histology. At the end of the follow up period, frozen sections of pancreas were stained with standard hemato-eosin saffron (HES) trichrome. The architecture of the organ was found to be preserved, with no sign of fibrosis or inflammation. (Insets: beta cell islets).

Tumor growth curve was compared from the day of the first injection (day 0) until the last day when all 40 mice remained included in the study (day 26) (Figure 4A,B). Tumor volumes from both 400 pmol and 800 pmol group were found to be significantly reduced in comparison to control group (*p* = 0.0001 and *p* = 0.0066, respectively) (Figure 4A). Moreover, relative tumor volume of 400 pmol group was found to be lower than that observed in the 800 pmol group (*p* = 0.0002) (Figure 4A). At later time points, some control mice tumors reached the limit size volume of 1500 mm^3^ and mice were therefore euthanized, whereas no tumor from the two treated groups reached this limit (Figure 4B, and Figure S1 for individual curves). Despite the fact that [^177^Lu]Lu-NeoB was no longer administered, a tumor regression was observed in 400 pmol and 800 pmol groups. From day 65, no residual tumor volume was found on any mice from the 400 pmol group. At the same time point (65 days) in the 800 pmol group 4 mice out of 13 had measurable tumors. This more pronounced effect of [^177^Lu]Lu-NeoB on tumor regression observed on 400 pmol in comparison to 800 pmol group is in accordance with the higher [^177^Lu]Lu-NeoB uptake in GRPR tumors (Figure 3). The efficacy of [^177^Lu]Lu-NeoB can also be represented using a survival graphic representation (Figure 4C). In this graphic it can be seen that the tumors in 5 out of 13 control mice reached the limit size during the 100 days period of follow-up, while none of the 400 pmol or 800 pmol mice were excluded due to an over limit tumor volume. No change was observed in total body weight of treated animals (Figure 4D), and no off-target effect, such as inflammation, fibrosis, or architecture alteration, was observed on HES stained pancreatic sections (Figure 5).

## 4. Discussion

The advent of radio-ligand therapies has opened new opportunities for the management of cancer patients and several compounds are currently in clinical development such as prostate-specific membrane antigen targeting peptides [11,29]. The overexpression of GRPR in several cancers makes it an attractive candidate for imaging and targeted therapy [4]. GRPR remains expressed at high level in lymph-nodes or distant metastases allowing the follow-up of the spread of the disease [20,30,31]. Most of GRPR-targeting radioligands developed for the diagnosis and therapy of GRPR-expressing tumors are analogues of amphibian bombesin [21,23,24]. A dozen clinical trials have been published so far, using GRPR-antagonists radiolabeled with Ga-68, Cu-64 and F-18 for diagnostic purpose. Most exhibited biosafety and have shown high diagnostic value in breast and prostate cancer [18,21,24]. Moreover, a clinical trial has also recently been performed to assess the dosimetry and safety of the GRPR-antagonist RM2 labeled with Lu-177 for therapy of metastatic prostate cancer [18]. Despite the low number of patients included (*n* = 4), it suggested that the absorbed doses in tumor and pancreas (1.076 ± 0.438 Gy/GBq) were compatible with therapeutic approach requirements. Indeed, when moving from diagnosis to therapeutic approaches with GRPR-antagonists, off-target binding in the pancreas that constitutively express GRPR may constitute a major bottleneck [32].

NeoB, a GRPR antagonist, has previously been successfully radiolabeled with Ga-67, Ga-68, In-111 and Lu-177. Biodistribution studies using NeoB have shown high tumor uptake with elevated tumor to background ratio in preclinical models of breast and prostate cancers, as well as elevated blood stability up to 30 min post-injection [22,23,24]. Following these promising results, the first in human clinical application with [^68^Ga]Ga-NeoB was published. [^68^Ga]Ga-NeoB strongly visualized the prostate primary tumor but also metastatic foci such as liver and bone metastases, with high lesion to background ratio [24]. Furthermore, a recent Phase I/IIa clinical study was concluded assessing the safety, tolerability, pharmacokinetics and dosimetry of [^68^Ga]Ga-NeoB in GIST patients (MITIGATE clinical trial, NCT02931929) [33]. Another important pre-clinical finding was the fact that the injected mass of NeoB constitutes a key parameter since it impacts the retention in off-target organs and therefore the dosimetry. As a matter of fact, Dalm et al. demonstrated that, when injected at an amount of 200 pmol in a mouse model of prostate cancer, [^177^Lu]Lu-NeoB exhibited a more favorable pharmacokinetic profile than when injected at an amount of 10 pmol, due to a lower pancreas retention [23]. Nevertheless, the therapeutic effect of NeoB has never been investigated.

In this study, we evaluate for the first time the theragnostic potential of [^177^Lu]Lu-NeoB for tumor treatment by performing biodistribution and therapeutic studies in mice bearing GIST. In accordance with above mentioned finding, injected mass ranging from 200 to 800 pmol were employed. Biodistribution study demonstrated high tumor uptake and retention in comparison to rapid elimination from the pancreas, resulting in elevated tumor-to-pancreas ratio (>300 at 1 week). These results are in accordance with previous biodistribution studies achieved with [^177^Lu]Lu-NeoB [23] Indeed, in PC-3 bearing mice, Dalm et al. obtained a tumor-to-pancreas ratio > 1 as early as 4 h after injection of 200 pmol of [^177^Lu]Lu-NeoB [23]. They observed a tumor half-life in prostate tumors of 28 h that is comparable to that obtained in the present study in GIST (40 h), and a pancreas half-life of 11 h (vs. 2.4 h in the present study). Therefore, the pharmacokinetic profile of [^177^Lu]Lu-NeoB in GIST bearing mice was at least comparable, if not more favorable, than that previously reported. [^177^Lu]Lu-NeoB biodistribution in mice has also been evaluated at three time points in a recent study dedicated to compare its biodistribution to that of [^177^Lu]Lu-ProBOMB1 in mice bearing prostate tumors [34]. Unlike what observed in the present study, pancreas-to-tumor ratios < 1 were obtained. This discrepancy can most likely be attributed to the difference in injected mass, thereby again emphasis on the importance of this parameter for therapeutic applications. In comparison to other GRPR-targeting agents, [^177^Lu]Lu-NeoB favorably compares with GRPR-radioagonists [^177^Lu]Lu-AMBA and [^177^Lu]Lu-BBN8, which exhibited tumor-to-pancreas and tumor-to-gastro-intestinal tract ratio < 1 at 24 h p.i [35]. [^177^Lu]Lu-NeoB biodistribution profile was similar to that obtained with [^177^Lu]Lu-RM2 and [^177^Lu]Lu-JMV4168 [36,37].

During the efficacy sub-study, two different peptide amounts, 400 pmol and 800 pmol, were compared. Tumor volumes from both treated groups were found to be significantly reduced in comparison to control group, with lower volumes obtained in the 400 pmol group. Interestingly, no residual tumor was found on any mice from this group. The [^177^Lu]Lu-NeoB effects on tumor volumes are in accordance with the higher tumor uptake of [^177^Lu]Lu-NeoB in the 400 pmol group in comparison to the 800 pmol group, observed on SPECT-CT images. This difference in tumor accumulation might be attributed to the reach of the maximal specific binding (Bmax) in the 400 pmol group. The results presented here are in line with previous results obtained with [^177^Lu]Lu-NeoB and [^177^Lu]Lu-AMBA [23,35]. While they have not been evaluated in the present study, biological mechanisms underlying the therapeutic effect of such ^177^lu-labeled ligands directed at tumor membrane antigens are well known. DNA damages can include not only double-strand breaks, but also single-strand breaks in proximity to base lesions, leading to the activation of apoptosis pathways [38].

Regarding the [^177^Lu]Lu-NeoB therapeutic potential here, it seems to be at least similar if not better than others ^177^Lu-radiolabeled GRPR antagonists, such as [^177^Lu]Lu-JMV4168, which was evaluated in PC-3 tumor bearing mice. After four cycles of injections (50 MBq, 200 pmol) in a two-days interval, a regrowth of tumors was present that was not observed in our study after three cycles of therapy in a one week interval (37 MBq for 400 or 800 pmol) [36]. Together, these results demonstrated that [^177^Lu]Lu-NeoB is a promising therapeutic agent for GRPR-expressing tumors.

## 5. Conclusions

[^177^Lu]Lu-NeoB exhibited excellent pharmacokinetics with elevated and prolonged tumor uptake and low uptake in non-target organs such as pancreas. At therapeutic doses, [^177^Lu]Lu-NeoB successfully inhibited GIST-882 tumor growth, leading to complete tumor regression at 400 pmol dose. Clinical trial is ongoing to confirm the potential of this new theragnostic agent in adult patients with advanced solid tumors known to overexpressed GRPR (NCT03872778).

## Data Availability

The data presented in this study are available on request from the corresponding author.

## Supplementary Materials

The following are available online at https://www.mdpi.com/2072-6694/13/5/1051/s1, Figure S1: Individual tumor growth curves from efficacy sub-study, Table S1: Biodistribution of ^177^Lu-NeoB on GRPR-tumor bearing mice.
