# Supplementary material for: In Vivo Biodistribution and Efficacy Evaluation of NeoB, A Radiotracer Targeted to GRPR, in Mice Bearing Gastrointestinal Stromal Tumor
Source: Cancers (Basel). 2021 Mar 2;13(5):1051. doi: 10.3390/cancers13051051 (PMC7958597; doi:10.3390/cancers13051051)
Supplement: Supplementary file 1 [file cancers-13-01051-s001.pdf]

# Supplementary Materials: In Vivo Biodistribution and Efficacy Evaluation of NeoB, a Radiotracer Targeted to GRPR, in Mice Bearing Gastrointestinal Stromal Tumor

**Christopher Montemagno, Florian Raes, Mitra Ahmadi, Sandrine Bacot, Marlène Debiossat, Julien Leenhardt, Jean Boutonnat, Francesca Orlandi, Donato Barbato, Mattia Tedesco, Catherine Ghezzi, Pascale Perret and Alexis Broisat**

**Table S1. Biodistribution of [$^{177}$Lu]Lu-NeoB on GRPR-tumor bearing mice.** Biodistributions were performed at 1 h, 4 h, 24 h, 48 h, 96 h, 168 h and at 4 h when co-injected with an excess of unlabeled NeoB (4h + block). Results are expressed in mean ± SD.

| | **1 h** | | | **4 h** | | | **24 h** | | | **48 h** | | | **96 h** | | | **168 h** | | | **4 h + block** | | |
|---|---|---|---|---|---|---|---|---|---|---|---|---|---|---|---|---|---|---|---|---|---|
| | **mean** | **SD** | ***n*** | **mean** | **SD** | ***n*** | **mean** | **SD** | ***n*** | **mean** | **SD** | ***n*** | **mean** | **SD** | ***n*** | **mean** | **SD** | ***n*** | **mean** | **SD** | ***n*** |
| **Adrenals** | 5.5 | 2.0 | 5 | 1.5 | 0.5 | 4 | 0.8 | 0.3 | 5 | 0.3 | 0.2 | 6 | 0.2 | 0.2 | 5 | 0.2 | 0.1 | 5 | 0.3 | 0.1 | 5 |
| **Bladder** | 20.6 | 19.7 | 4 | 3.1 | 3.8 | 5 | 0.1 | 0.1 | 5 | 0.1 | 0.1 | 6 | 0.0 | 0.0 | 5 | 0.0 | 0.0 | 5 | 5.9 | 8.3 | 5 |
| **Blood** | 2.9 | 0.2 | 5 | 0.2 | 0.0 | 5 | 0.0 | 0.0 | 5 | 0.0 | 0.0 | 6 | 0.0 | 0.0 | 5 | 0.0 | 0.0 | 5 | 0.1 | 0.0 | 5 |
| **Bone** | 0.7 | 0.3 | 5 | 0.1 | 0.0 | 5 | 0.0 | 0.0 | 5 | 0.0 | 0.0 | 6 | 0.0 | 0.0 | 5 | 0.0 | 0.0 | 5 | 0.1 | 0.0 | 5 |
| **GIST tumor** | 21.6 | 1.7 | 5 | 19.1 | 3.9 | 5 | 13.4 | 3.5 | 5 | 10.5 | 1.6 | 6 | 5.9 | 0.3 | 5 | 2.2 | 0.7 | 5 | 0.3 | 0.1 | 5 |
| **Heart** | 1.0 | 0.2 | 5 | 0.1 | 0.0 | 5 | 0.0 | 0.0 | 5 | 0.0 | 0.0 | 6 | 0.0 | 0.0 | 5 | 0.0 | 0.0 | 5 | 0.1 | 0.0 | 5 |
| **Kidneys** | 6.1 | 0.3 | 5 | 1.8 | 0.4 | 5 | 0.4 | 0.1 | 5 | 0.1 | 0.0 | 6 | 0.1 | 0.0 | 5 | 0.0 | 0.0 | 5 | 2.0 | 0.6 | 5 |
| **Large bowel** | 2.8 | 0.6 | 5 | 2.0 | 0.4 | 5 | 0.4 | 0.2 | 5 | 0.1 | 0.0 | 6 | 0.1 | 0.0 | 5 | 0.0 | 0.0 | 5 | 0.6 | 0.6 | 5 |
| **Large bowel content** | 1.1 | 1.2 | 5 | 10.0 | 3.5 | 5 | 1.3 | 1.0 | 5 | 0.4 | 0.3 | 6 | 0.4 | 0.4 | 5 | 0.3 | 0.2 | 5 | 10.0 | 8.8 | 5 |
| **Liver** | 5.8 | 1.1 | 5 | 0.7 | 0.1 | 5 | 0.3 | 0.1 | 5 | 0.2 | 0.0 | 6 | 0.2 | 0.0 | 5 | 0.1 | 0.0 | 5 | 0.6 | 0.1 | 5 |
| **Lungs** | 2.3 | 0.4 | 5 | 0.2 | 0.1 | 5 | 0.0 | 0.0 | 5 | 0.1 | 0.1 | 6 | 0.0 | 0.0 | 5 | 0.0 | 0.0 | 5 | 0.2 | 0.1 | 5 |
| **Pancreas** | 19.8 | 2.6 | 5 | 8.5 | 2.0 | 5 | 1.1 | 0.4 | 5 | 0.2 | 0.0 | 6 | 0.0 | 0.0 | 5 | 0.0 | 0.0 | 5 | 0.1 | 0.0 | 5 |

| | | | | | | | | | | | | | | | | | | | | | |
|---|---|---|---|---|---|---|---|---|---|---|---|---|---|---|---|---|---|---|---|---|---|
| **Small bowel** | 3.9 | 1.0 | 5 | 1.8 | 0.7 | 5 | 0.3 | 0.1 | 5 | 0.1 | 0.0 | 6 | 0.0 | 0.0 | 5 | 0.0 | 0.0 | 5 | 0.3 | 0.1 | 5 |
| **Small bowel content** | 5.1 | 2.9 | 5 | 0.8 | 0.3 | 5 | 0.1 | 0.1 | 5 | 0.0 | 0.0 | 6 | 0.0 | 0.0 | 5 | 0.0 | 0.0 | 5 | 0.4 | 0.2 | 5 |
| **Spleen** | 1.2 | 0.2 | 5 | 0.3 | 0.1 | 5 | 0.1 | 0.1 | 5 | 0.1 | 0.0 | 6 | 0.0 | 0.0 | 5 | 0.1 | 0.1 | 5 | 0.3 | 0.1 | 4 |
| **Skeletal muscle** | 0.4 | 0.0 | 5 | 0.1 | 0.0 | 5 | 0.0 | 0.0 | 5 | 0.0 | 0.0 | 6 | 0.0 | 0.0 | 5 | 0.0 | 0.0 | 5 | 0.0 | 0.0 | 5 |
| **Stomach** | 3.7 | 1.0 | 5 | 1.9 | 0.6 | 5 | 0.7 | 0.2 | 5 | 0.3 | 0.1 | 6 | 0.1 | 0.1 | 5 | 0.0 | 0.0 | 5 | 0.2 | 0.1 | 5 |
| **Urine** | 600.2 | 155.4 | 5 | 215.5 | 192.9 | 5 | 1.9 | 1.1 | 5 | 0.9 | 0.5 | 6 | 0.3 | 0.1 | 5 | 0.2 | 0.2 | 5 | 116.4 | 146.8 | 5 |

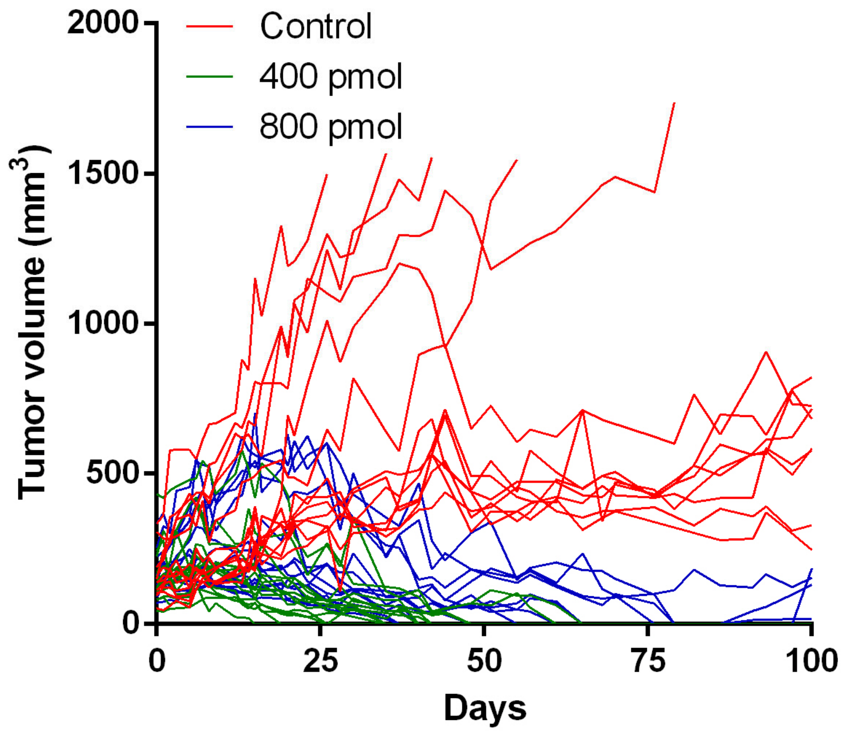


**Figure S1.** Individual tumor growth curves from efficacy sub-study. Tumor volumes were determined up to 100 days following the onset of the therapy. Mice with tumor reaching 1500mm$^3$ were excluded from the study. Data are expressed in mm$^3$.
